# Enhanced photocatalytic activity of phosphorene under different pH values using density functional theory (DFT)

**DOI:** 10.1039/d0ra10246f

**Published:** 2021-04-29

**Authors:** Mamori Habiba, Benyoussef Abdelilah, El Kenz Abdallah, Taleb Abdelhafed, Ahmed Ennaoui, El Maalam Khadija, Mounkachi Omar

**Affiliations:** Laboratory of Condensed Matter and Sciences Interdisciplinary (LaMCScI), Faculty of Science, Mohammed V University in Rabat BP 1014 RP Rabat Morocco omar.mounkachi@um5.ac.ma o.mounkachi@um5r.ac.ma; Materials and Nanomaterials Center, Moroccan Foundation for Advanced Science, Innovation and Research, MAScIR Rabat Morocco; PSL Research University, Chimie ParisTech – CNRS, Institut de Recherche de Chimie Paris 75005 Paris France; Sorbonne University 4 Place Jussieu 75231 Paris France; Scientific Council of IRESEN, the Moroccan Solar Energy Research Institute Ben Guerir Morocco; MSDA, Mohammed VI Polytechnic University Lot 660, Hay Moulay Rachid Ben Guerir 43150 Morocco; Hassan II Academy of Sciences and Techniques Rabat Morocco

## Abstract

Phosphorene, a new two-dimensional material, was investigated theoretically as a promising photocatalyst material. The structural and electronic properties of phosphorene were studied using hybrid functional based HSE approximation. The effect of the adsorbed molecules on the phosphorene surface was studied for various chemical elements, such as water molecule (H_2_O), hydronium ion (H_3_O^+^), hydrogen atom and ion (H/H^+^), hydroxide molecule (OH), and hydroxide ion (OH^−^). The potential application of phosphorene as a photocatalyst in vacuum was proved under different pH values. A pH of 8 was found to be the suitable value for clean phosphorene in which the flat band position was corrected for the oxidizing and reducing potentials of phosphorene, but the presence of OH^−^ ions in a basic solution damaged the surface structure and limited the use of phosphorene in photocatalysis caused by the high content (0.25 ML and 0.5 ML) of the adsorbed OH^−^ on the phosphorene surface. The obtained results matched the required parameters of a photocatalyst for water splitting using clean phosphorene surface in neutral solution (pH = 7).

## Abstract 2

Phosphorene, a new two-dimensional material, was investigated theoretically as a promising photocatalyst material.

## Graphical abstract

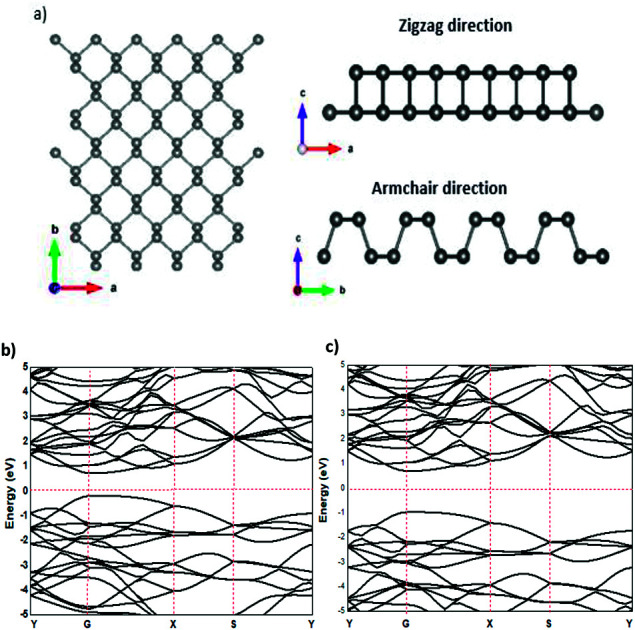

## 1. Introduction

A photocatalyst is a material that can absorb photons from sunlight and induce the formation of electron–hole pairs to initiate redox reactions for hydrogen production.^1,2^ A number of photocatalyst materials have been developed, and they can be divided into three generations. The first generation was based on metals with only ultraviolet absorption, the second generation also concerned metals but with visible light absorption and the third one was based on two-dimensional materials with visible light absorption. The first and second generations resulted in low hydrogen conversion, which induced a low photocatalytic efficiency, while the third generation remediated this issue due to its unique structural, electronic, and optical properties.^2^ Recently, a new two-dimensional material named phosphorene has been successfully synthesized from bulk black phosphorus structure.^3^ The material presents new and interesting structural,^4^ electronic^4^ and optical properties.^5^ Black phosphorus is the most stable structure of phosphorus than other allotropic forms like white, red, and violet phosphorus.^6^ The research on the potential application of single- or few-layer phosphorene has been demonstrated for several applications, such as photovoltaics,^7^ photocatalysis,^2,8^ electronics,^9^ photonics,^10^ and batteries.^11,12^ This wide range of applications, in contrast to those of graphene and transition-metal dichalcogenides, could be explained by the intrinsic structure, the mechanical anisotropy, and the electronic, optoelectronic and thermoelectric properties of phosphorene. These properties are related to its puckered structure, where the bond angle along the zigzag direction is 96.34 Å, and the adjacent P–P bond length is 2.224 Å. The corresponding dihedral angle along the zigzag direction is 103.09 Å with the connecting P–P bond length being 2.244 Å.

A few layers of phosphorene demonstrated a weak van der Waals interlayer interaction and a strong covalent bonding.^13^ The interlayer distance depended on the stacking order and could be adjusted from 3.21 Å to 3.73 Å,^14,15^ which facilitated the mechanical exfoliation of single- or few-layers of phosphorene. Phosphorene is a p-type semi-conductor material with a band gap dependent on the layer number and could be changed from 0.3 eV to 2 eV; this characteristic feature was named the quantum confinement effect, and it resulted in high absorption range.^16^ Although black phosphorus is the most stable allotrope of phosphorus, it is still unstable under ambient conditions due to the sp^3^ hybridization, which affects its applications under ambient conditions.^17,18^

In this theoretical work based on DFT calculations, we studied the effect of different adsorbed chemical elements, such as H, H^+^, OH, OH^−^, H_3_O^+^, and H_2_O, on the structural, electronic, and optical properties of one-layered phosphorene. In addition, the potential application of clean and adsorbed phosphorene as a photocatalyst in vacuum and at different pH was analysed in detail.

## 2. Computational methods

This work was based on density functional theory (DFT) using Quantum espresso code (PW),^19^ with 2 × 2 × 1 supercell using Perdew–Burke–Ernzerhof (PBE) approximation.^20^ The cell parameters and atomic positions were successfully relaxed to find the equilibrium structure. The structural convergence was achieved with a cutoff energy of 500 eV, *K*-points of 5 × 5 × 1 for structural relaxation, and 15 × 15 × 1 for calculation of properties. In order to prevent the interaction between the periodic images, we introduced a vacuum thickness of 12 Å in the *z*-axis. The relaxation convergence for the ions and electrons was about 1 × 10^−6^ eV. Based on the optimized structure, a self-consistent calculation was applied to extract the electronic properties using the norm conserving the pseudopotential and the projector augmented wave (PAW) functional.^21^ As is known, the exchange-correlation (XC) functionals in the local DFT methods like the generalized gradient approximation (GGA)^22,23^ significantly underestimate the band gap (*E*_g_) due to the existence of a derivative discontinuity of the energy with respect to the number of electrons in the system. To substantially improve the band gap value, we included 25% of the exact Hartree–Fock (HF) exchange in the Coulomb potential by using the screened hybrid functional of Heyd–Scuseria–Ernzerhof (HSE).^24^ The optical response for phosphorene was obtained by using the HSE functional and the time-dependent density functional theory (TDDFT). All of the atomic positions and lattice vectors were kept the same as those in the lowest energy structure found for GGA–PBE functional.

## 3. Results and discussion

**Fig. 1:**
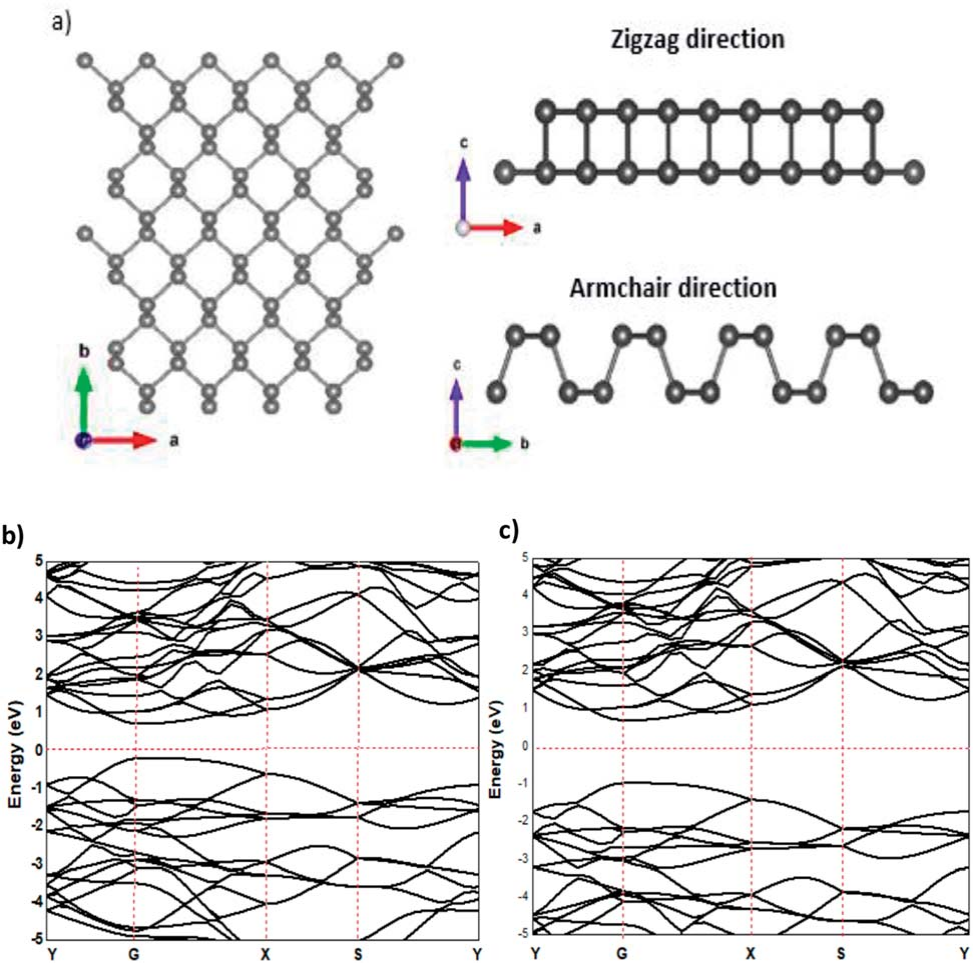
Structural properties (a) and the electronic band structure of phosphorene using GGA–PBE (b) and HSE (c) functionals.

The relaxed phosphorene structure obtained using the GGA–PBE approximation has an orthorhombic crystal structure with the space group *Cmca* (64); each P atom is directly bonded to the three other neighbouring atoms *via* covalent bonds as shown in Fig. 1(a and b), where each atom has two neighbours at 2.224 Å and a third one at 2.26 Å. The structure of phosphorene is puckered, resulting from the remaining lone pairs on each P atom, having a single-layer with a honeycomb-like lattice with bond angles of 96.34° and 103.09°, which is consistent with the ref. 25 The *x*-axis referred to the zigzag direction, whereas the *y*-axis referred to the armchair direction. The band gaps obtained using the GGA–PBE and HSE functionals were 0.9 eV (Fig. 1(b)) and 1.66 eV (Fig. 1(c)), respectively, which are in good agreement with other theoretical works.^26,27^

### 3.1. Phosphorene adsorbed molecules and ions

**Fig. 2:**
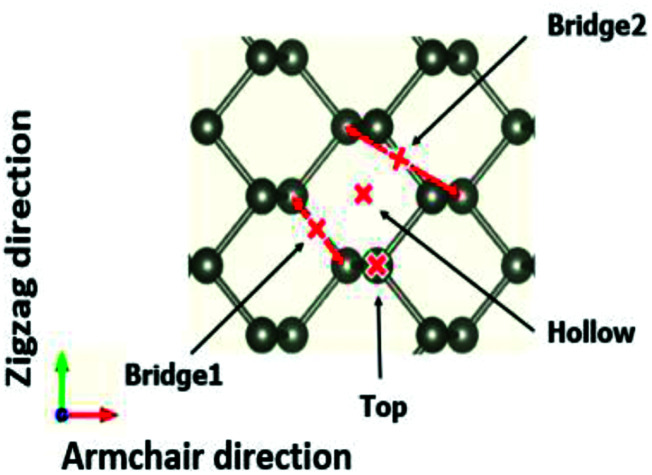
Four different adsorption sites on the phosphorene surface, namely the top, bridge1, bridge2, and hollow sites.

The adsorption structures of molecules and ions at different phosphorene sites were investigated. In our calculations, four different adsorption sites on the phosphorene surface were modelled: the top site, the hollow site, the bridge1 site, and the bridge2 site. We then examined the energetic and thermodynamic stability of the adsorbed molecules and ions on the stable site by calculating the cohesive energy per unit area at different concentrations and the adsorption energy per unit area^28^Fig. 2.*E*_coh_ = [*E*_slab+Ad_ − *n*_P_*E*_P_ − *n*_Ad_*E*_Ad_]/*S*,*E*_ad_ = [*E*_slab+Ad_ − *E*_slab_ − *n*_Ad_*E*_Ad_)]/*S*.Here, *n*_P/Ad_ is the total number of P atoms and adsorbed adatoms, molecules or ions in the system, *E*_P/Ad_ is the total energy of the isolated P atom and the adsorbed adatoms, molecules or ions, *S* is the area of the surface, *E*_slab+Ad_ and *E*_slab_ are the total energies of the phosphorene surface with the adsorbed molecules or ions and the clean phosphorene surface, respectively. The calculated energy required to break the chemical bonds between the phosphorene surface and the adsorbed adatoms, molecules or ions was defined as the binding energy (*E*_b_):^29^*E*_b_ = − [*E*_slab+Ad_ − *E*_slab_ − *E*_Ad_)]

The calculated *E*_coh_ and *E*_Ad_ are presented in Table 1 for the adsorbed molecules and ions at the most stable site on the phosphorene surface at different concentrations. The *E*_Ad_ of these systems are all negative, which indicates that the structures are stable, except in the case of OH^−^ ions where a small positive value is shown to indicate that the adsorption process is exothermic. Moreover, it can be seen from Table 1 that the absolute adsorption and cohesive energy increase as the surface coverage increases from 0.0625 mL to 0.5 mL, which reflects that the structural stability of the adsorbed molecules and ions can be improved by increasing the surface coverage. The energy required for breaking the p–H^+^ bond was found to be the most energetic, while p–H_2_O presented the lowest binding energy due to the weak interaction.

**Table 1:** Adsorption energy and surface cohesive energy for different molecules and ions adsorbed on the phosphorene surface

Systems		H	H^+^	OH	OH^−^	H_2_O	H_3_O^+^
Adsorption site		Top	Top	Top	Top	Hollow	Bridge 2
*E*_ad_ (eV Å^−2^)	0.0625 ML	−0.0038	−0.0249	−0.0035	0.0041	−0.0001	−0.0117
	0.25 ML	−0.0173	−0.0921	−0.0167	0.0194	−0.0025	−0.0553
	0.5 ML	−0.0392	−0.1629	−0.0388	0.0401	−0.0064	−0.0897
*E*_coh_ (eV Å^−2^)	0.0625 ML	−0.1514	−0.1725	−0.1511	−0.1435	−0.1477	−0.1593
	0.25 ML	−0.1650	−0.2397	−0.1643	−0.1282	−0.1501	−0.2029
	0.5 ML	−0.1868	−0.3105	−0.1864	−0.1075	−0.1541	−0.2373
*E*_b_ (eV)	0.0625 ML	2.0108	13.1766	1.8521	2.1696	0.0529	6.1914

**Fig. 3:**
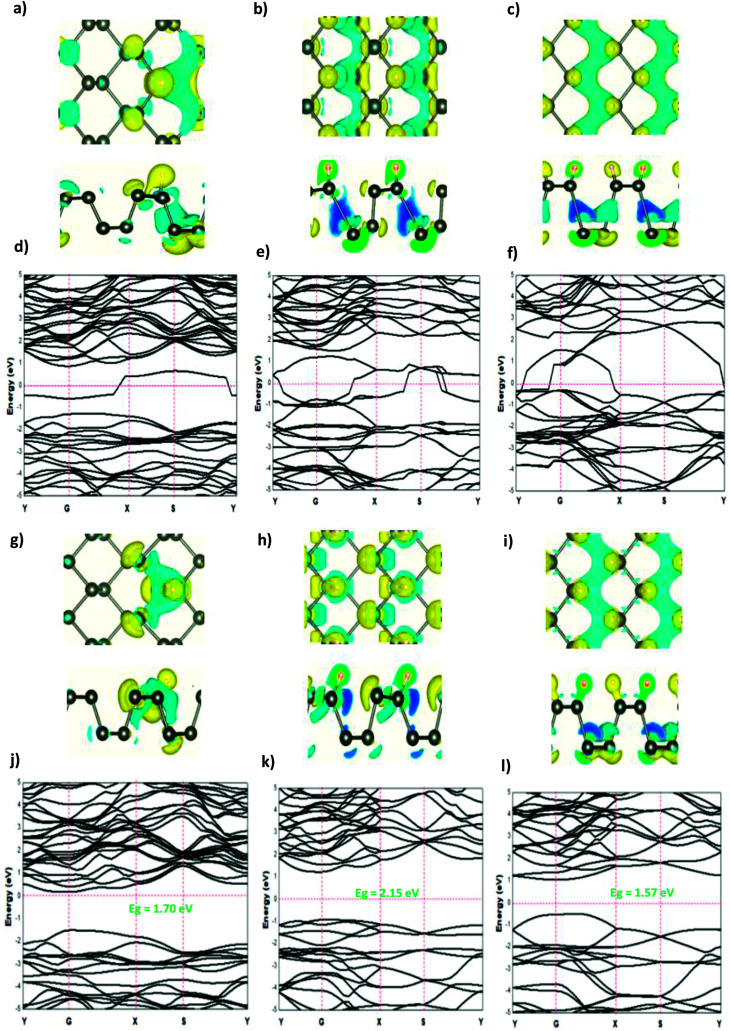
The charge density difference of H and H^+^ at a concentration at (a and g) 0.0625 ML, (b and h) 0.25 ML, and (c and i) 0.5 ML, respectively, on the phosphorene surface; the band structure of H and H^+^ at (d and j) 0.0625 ML, (e and k) 0.25 ML, and (f and l) 0.5 ML, respectively.

The modified surface structure could significantly change the electronic properties of phosphorene by forming new bonds at the surface of phosphorene.^16,21,25^ In order to analyze the adsorption effect of the adatoms and ions on the phosphorene surface, we first studied the charge density difference for the H adatom and the H^+^ ion adsorbed on the phosphorene surface (Fig. 3). For H (Fig. 3(a)–(c)), the charge density is mainly shared by the H adatom and the P atom to form covalent bonds. Moreover, it could be seen that the H atom had a strong interaction with the P atoms, which resulted from the charge accumulation between the H adatoms and P atoms. For H^+^ ion adsorption, the results indicate that there is a remarkable charge transfer from the phosphorene valence band to H^+^ ions, which led to the phosphorus–H^+^ coordination bond feature. As the concentration increased from 0.0625 mL to 0.5 mL, the vdW interaction between the two planes of the phosphorene monolayer became weak due to the increase in the distance during the formation of covalent bonds between the H adatoms and P atoms, while in the case of H^+^ ions the structure of phosphorene was not perturbed. To illustrate the electronic structure of the H adatoms and H^+^ ions adsorbed on phosphorene, the band structure is presented in Fig. 3. The H adatom with all of the atoms and concentrations studied (0.0625 mL, 0.25 mL, and 0.5 mL) caused a metallic transition in the semi-conductor nature of phosphorene, which resulted from the addition of new bonds in the band gap region, whereas during the addition of H^+^ ions, the semi-conductor nature of phosphorene was maintained; moreover, the Fermi level shift to the valence band with an increase in the concentration of H^+^ ions resulted in a p-type doping, thereby making H^+^ ion an acceptor (Fig. 3). As a result of the adsorption of H_3_O^+^ ions and H_2_O molecule on the phosphorene surface, the H_3_O^+^ ion decomposed and formed H^+^ ions that were adsorbed on the surface, and the H_2_O molecule was located close to the adsorbed H^+^ ion. This geometry supposed that the H_2_O molecule was attracted to the adsorbed H^+^ ion to form an H-bond between the O atom and the H^+^ ion. As the concentration increased, the H_2_O molecule desorbed from the phosphorene surface. For 0.5 mL concentration of the adsorbed H_3_O^+^ ions, some H^+^ ions were shown to detach from the phosphorene surface. However, it caused a shift in the Fermi level into the valence bands and formed a p-type doping, thereby conserving the phosphorene structure. The adsorption of the H_2_O molecule on the surface of phosphorene caused charge depletion from the H_2_O molecule and charge accumulation on the P atoms with a large charge redistribution that resulted from the large dipole moment of the H_2_O molecule. This prominent effect does not substantially change the electronic structure, but it moved the Fermi level to the conduction bands as the concentration increased, thereby causing an n-type doping, which resulted from the donor nature of the H_2_O molecule on the surface of phosphorene.

**Fig. 4:**
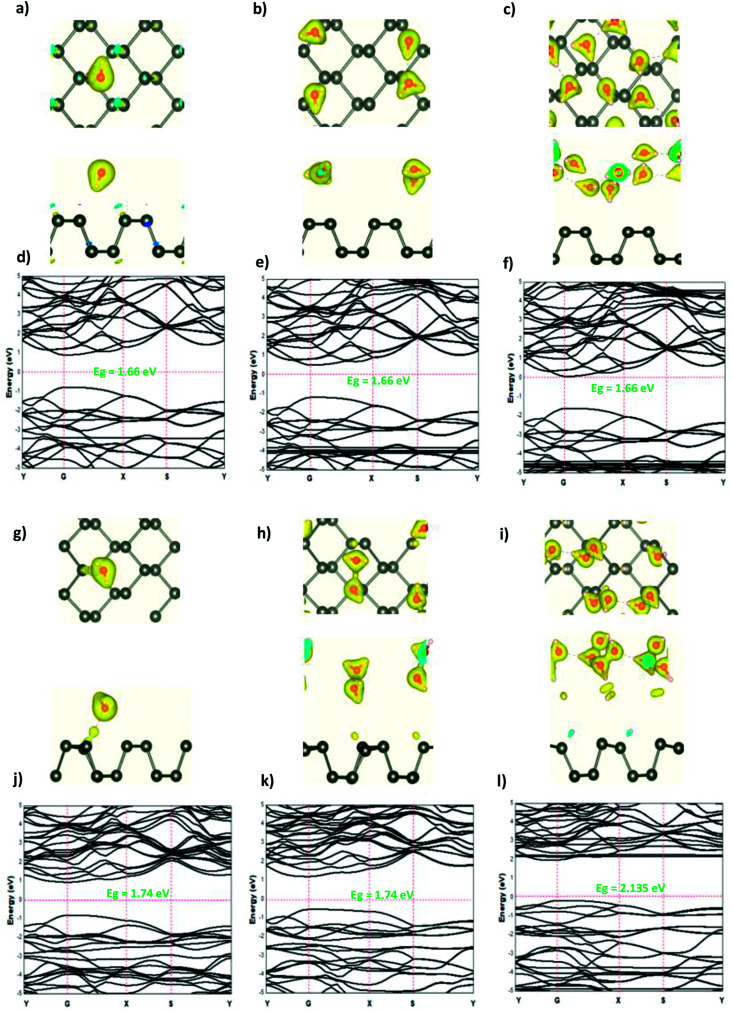
The charge density difference of H_2_O and H_3_O^+^ at a concentration at (a and g) 0.0625 ML, (b and h) 0.25 ML, and (c and i) 0.5 ML, respectively, on the ML phosphorene surface; the band structure of H_2_O and H_3_O^+^ at (d and j) 0.0625 ML, (e and k) 0.25 ML, and (f and l) 0.5 ML, respectively.

**Fig. 5:**
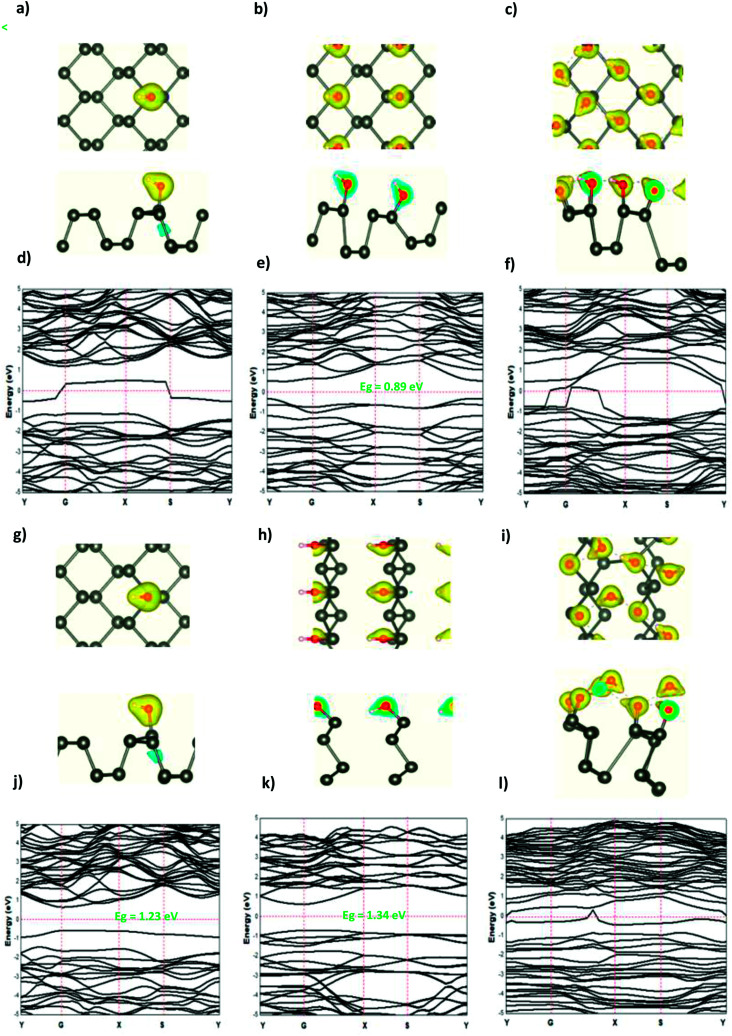
The charge density difference of OH and OH^−^ at a concentration at (a and g) 0.0625 ML, (b and h) 0.25 ML, and (c and i) 0.5 ML, respectively, on the ML phosphorene surface; the band structure of OH and OH^−^ at (d and j) 0.0625 ML, (e and k) 0.25 ML, and (f and l) 0.5 ML, respectively.

The adsorption of the OH molecules and OH^−^ ions on the surface of phosphorene resulted in charge accumulation in the OH molecule and OH^−^ ion, whereas a loss of electrons in phosphorene. This charge transfer caused a distortion of the phosphorene structure as suggested by the orbital hybridization between the O and the P atoms. The electronic structure illustrated that at a low OH concentration (0.0625 ML), the orbitals of the OH molecule were located in the band gap region and created defect states in the ML phosphorene. As the concentration was increased to 0.25 ML, the band gap of phosphorene was reduced to 0.89 eV with a direct nature at the *Y* point. For 0.5 ML concentration, the orbital hybridization between the P atoms and the OH molecules induced the transition from the semi-conductor to the metallic nature. In the case of OH^−^ ions at a low concentration (0.0625 ML), the semi-conductor nature of phosphorene was maintained but the band gap decreased, which was caused by the defect states in the band gap region; however, the structure of phosphorene was destroyed forming new nanostructures at 0.25 ML. The semi-conductor with a direct band gap (1.34 eV) at G point was observed. For 0.5 ML concentration, the semi-conductor became metallic in nature (Fig. 4 and 5).

From all of the above, it was expected that the adsorbed molecules, ions, and adatoms on the surface of phosphorene resulted in a band gap reduction, especially in the case of H_3_O^+^ (0.0625 ML, 0.25 ML), OH (0.0625 ML, 0.25 ML, and 0.5 ML), OH^−^ (0.0625 ML, 0.25 ML, and 0.5 ML), H (0.0625 ML, 0.5 ML), and H^+^ (0.5 ML), owing to the defect states present inside the band gap. In the case of water adsorption, the band gap of phosphorene was maintained. These defect states are favorable for electron transport, hence improving the electron transport capability and increasing the phosphorene light absorption efficiency.

### 3.2. Optical properties

**Fig. 6:**
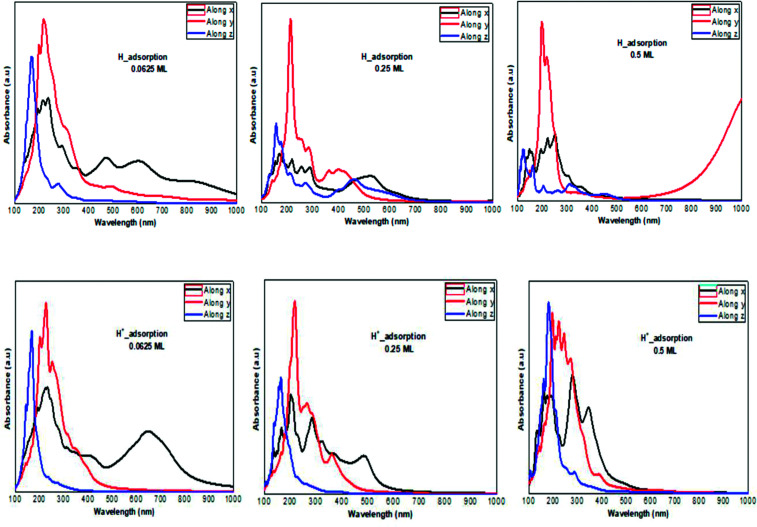
The calculated optical absorption curves of H molecules and H^+^ ions adsorbed on ML phosphorene surface at different concentrations along the three directions.

**Fig. 7:**
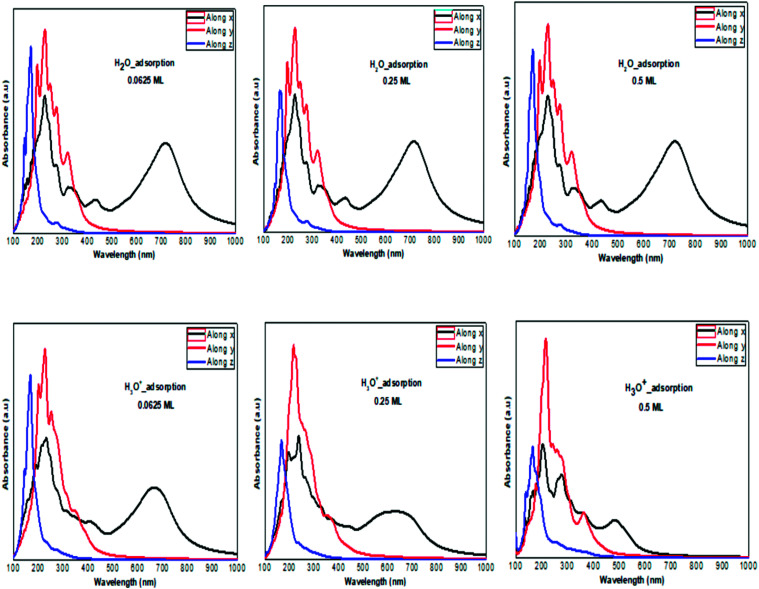
The calculated optical absorption curves of H_2_O molecules and H_3_O^+^ ions adsorbed on ML phosphorene surface at different concentrations along the three directions.

**Fig. 8:**
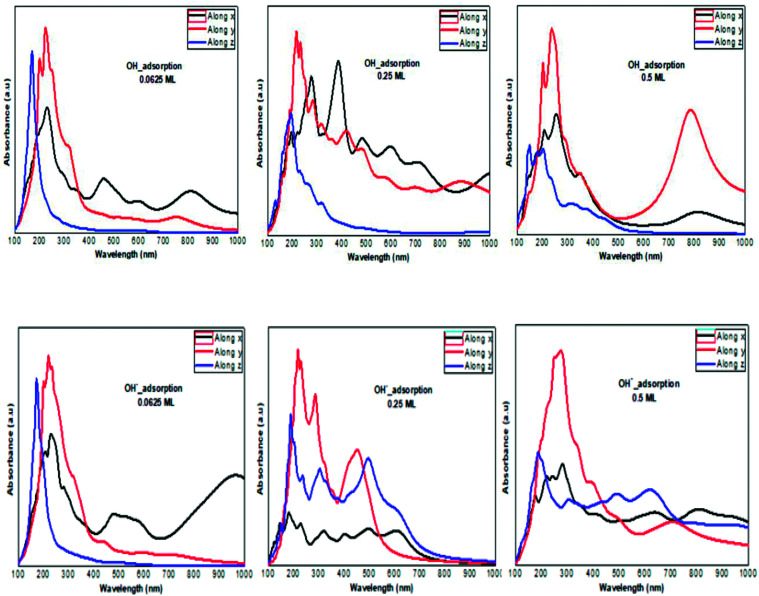
The calculated optical absorption curves of OH molecules and OH^−^ ions adsorbed on phosphorene surface at different concentrations along the three directions.

**Fig. 9:**
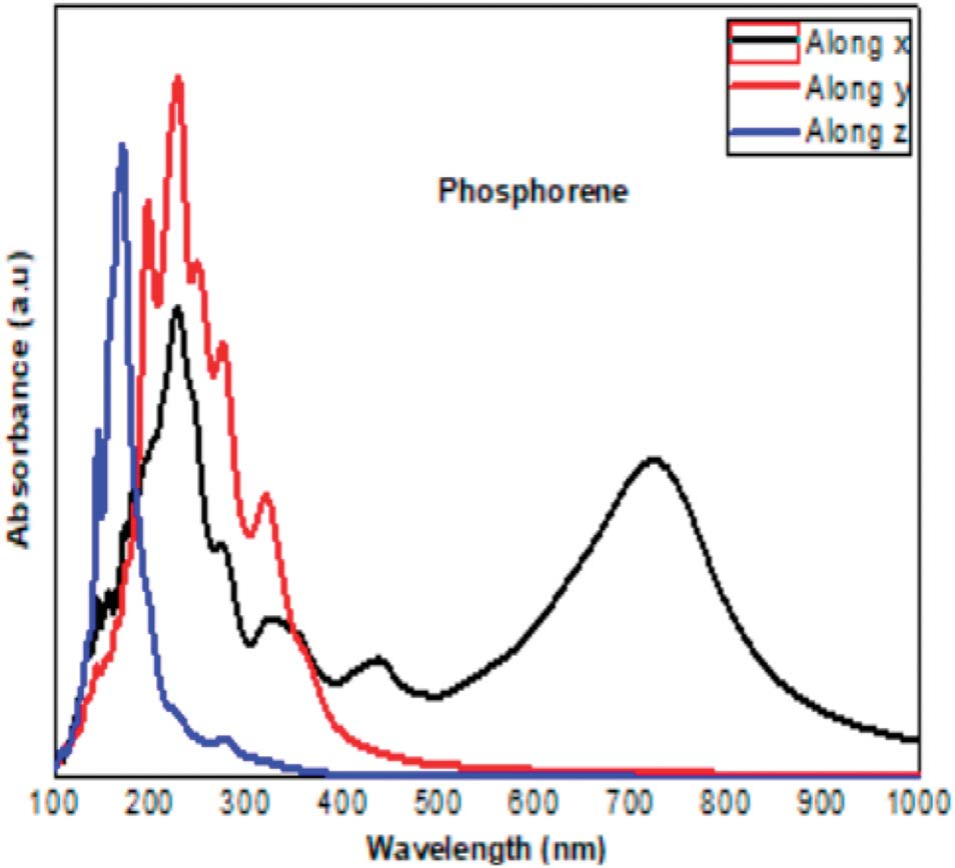
The calculated optical absorption curve of clean phosphorene along the three directions, in agreement with the experimental results obtained at ambient conditions.^30^

The optical absorption response for the H adatom adsorption (Fig. 6) induced changes in the optical response of phosphorene (Fig. 9) due to the defect states formation in the band gap, which caused the absorption limit to shift to the infrared region. For H^+^ ions, the optical absorption maintained the visible absorption response with a good coverage of the visible light spectra at a low concentration (0.0625 ML). The calculated optical properties of the H_2_O molecules and H_3_O^+^ ions adsorbed on the phosphorene surface are shown in Fig. 7. The optical absorption curves indicated that the adsorption of H_2_O molecule had no effect on the optical absorption of phosphorene (Fig. 9). The H_3_O^+^ ion demonstrated negligible changes at 0.0625 ML concentration; at 0.25 ML concentration, the system maintained an absorption curve in the visible region (around 650 nm) and at 0.5 ML concentration, the system had an absorption curve at 500 nm, which suggested that the systems with both H_2_O and H_3_O^+^ adsorption maintained a positive light response. The optical response for OH molecule and OH^−^ ion adsorption on phosphorene showed a good coverage of the visible light spectra (400–800 nm). As the concentration increased, the absorption response became more significant, and this adsorption was a way to improve the optical properties of phosphorene (Fig. 8).

### 3.3. Photocatalytic properties

In order to evaluate the photocatalytic activities of the clean phosphorene and the adsorbed surface, we evaluated the relative position of the calculated band edge energy levels with respect to the water redox potential. In this calculation, the potential in the vacuum region was defined as the reference vacuum level. The voltage of the water splitting reaction was 1.23 V. The reduction and oxidation potentials of H^+^/H_2_ and O_2_/H_2_O with reference to the vacuum level^31^ are defined as follows (Fig. 9):*E*_H^+^/H_2__ = −4.44 + pH × 0.059 eV*E*_O_2_/H_2_O_ = −5.67 − pH × 0.059 eV

**Fig. 10:**
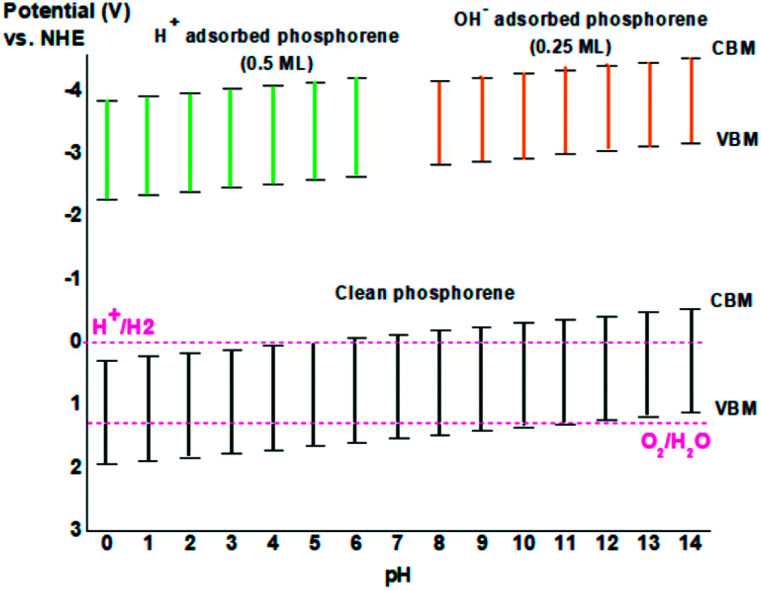
The band edges of clean phosphorene as a function of pH value, and phosphorene adsorbed ions H^+^ at 0.5 ML concentration and OH^−^ at 0.25 ML concentration.

The theoretical band edge values of the clean phosphorene and all of the molecules and ions adsorbed on the surface of phosphorene are illustrated in Fig. 10. The standard water reduction and oxidation at the point of zero charge solution was calculated as below:^32^*E*_VB_ = *χ*_p_ − *E*_0_ + 0.5 × *E*_g_*E*_CB_ = *E*_VB_ − *E*_g_where *χ*_p_, *E*_0_, and *E*_g_ are, respectively, the Mulliken electronegativity of atoms,^33^ the scaling factor relating the hydrogen electrode scale (NHE) to the absolute vacuum scale (AVS) (∼4.5 eV *vs.* AVS for 0 V *vs.* NHE), and the electronic band gap energy (1.66 eV) for phosphorene calculated using HSE approximation.

The CBM of phosphorene was located more negative than the redox potential of H^+^/H_2_ corresponding to the potential 0 V *vs.* NHE, while the VBM of phosphorene did not occupy a location more positive than the redox potential of O_2_/H_2_O corresponding to the potential 1.23 V *vs.* NHE (Fig. 10). As a result, phosphorene under vacuum conditions is not enough to produce hydrogen from photocatalytic water-splitting because of its too low band edge position. The presence of OH^−^ ions in basic solution and H^+^ ions in acidic solution could lead to the adsorbed H^+^ and OH^−^ ions on the surface of phosphorene, which could adjust the position of the band edge of clean phosphorene, thereby making it not suitable for hydrogen generation by photocatalytic water splitting at low or high ion concentration (Fig. 10). For clean phosphorene surface, and to equilibrate both the oxidizing and reducing potentials in phosphorene, the pH of the solution could shift the band edge alignment making it suitable to the redox potential of H^+^/H_2_ and O_2_/H_2_O. For instance, in a basic solution (pH = 8), the oxidation potential as well as the reduction potential shifted from the initial value making phosphorene appropriate for hydrogen production for photocatalytic application, but in the case of adsorbed OH^−^ ions on the surface of phosphorene, neutral solution with pH = 7 could be more appropriate for the use of phosphorene as a photocatalyst (Fig. 10).

## 4. Conclusion

This work presents a detailed theoretical study based on hybrid density functional theory calculation for the structural, electronic, and optical properties of phosphorene. The effect of adsorbed water molecule (H_2_O), hydronium ion (H_3_O^+^), hydrogen atom (H) and hydrogen ion (H^+^), hydroxide molecule (OH), and finally hydroxide ion (OH^−^) at different concentrations on the phosphorene surface demonstrated the formation of defect states on the clean phosphorene surface. For all of the molecule, ion, and adatom concentrations, the absolute adsorption energy increased when the surface coverage increased. In addition, the optical response showed that at a specific concentration, the phosphorene surface still maintained the positive optical response to visible light. The photocatalytic activity of phosphorene was very poor in vacuum, where this value was tuned to cover the oxidizing and reducing potentials at pH = 8. The presence of the adsorbed molecules, ions, and adatoms on the phosphorene surface resulted in changes in the band edge position of the clean phosphorene, thereby making it a bad photocatalyst material for water splitting to produce hydrogen. This study suggested the application of phosphorene as a photocatalyst in neutral solution (pH = 7) or to encapsulate the surface maintaining the electronic, optical, and photocatalytic activity of the clean phosphorene surface.

## Conflicts of interest

There are no conflicts to declare.

## Supplementary Material
